# Educational attainment and disease severity are associated with willingness to pay for rosacea treatment in Southwest China: a cross-sectional study

**DOI:** 10.3389/fpubh.2026.1878516

**Published:** 2026-10-01

**Authors:** Hongye Wu, Yanyan Feng, Qian Yao, Linli He, Jincui Shen, Jiayi Li, Qiao Chen, Yuanrong Tao

**Affiliations:** West China School of Medicine, Sichuan University, Sichuan University Affiliated Chengdu Second People’s Hospital, Chengdu, China

**Keywords:** cross-sectional study, educational attainment, rosacea, Southwest China, willingness to pay

## Abstract

**Background:**

Rosacea often requires long-term treatment and may impose substantial psychosocial and financial burden. However, evidence on willingness to pay (WTP) for Rosacea treatment and its socioeconomic and clinical correlates remains limited.

**Objective:**

To examine factors associated with WTP among rosacea patients in Southwest China, using a proportional affordability metric to distinguish socioeconomic capacity from disease-specific factors.

**Methods:**

In this cross-sectional study (2024–2025), 506 patients from a tertiary hospital completed surveys assessing WTP and Rosacea Quality of Life (RosaQoL), while clinical severity was rated by dermatologists. Univariate analysis (chi-square test) and binary logistic regression models were employed to examine factors associated with WTP among patients in Southwest China.

**Results:**

Among 506 rosacea patients (91.1% female, mean age 35.3 years), mean WTP was 25.3% of monthly income. In multivariable analysis, educational attainment was associated with high WTP after adjustment for income, with significantly lower odds among patients with junior high school education or below and senior high school education compared with those with postgraduate education or above; the university/college group showed a borderline association. Pairwise contrasts also suggested lower odds of high WTP among patients with mild or moderate rosacea than among those with severe rosacea; however, the overall Wald test for disease severity was borderline (*p* = 0.070), and the RosaQoL total score was not significant after adjustment.

**Conclusion:**

In Southwest China, educational attainment was the factor most consistently associated with WTP for rosacea treatment, whereas clinician-rated disease severity showed a severity-related pattern that should be interpreted cautiously given the borderline overall Wald test. As educational attainment may partly reflect health literacy and socioeconomic positioning, counseling should incorporate education-sensitive communication to support informed, affordability-aware treatment decisions.

## 1 Introduction

Rosacea is a common, chronic inflammatory dermatosis that typically presents with transient central facial flushing, persistent erythema, telangiectasias (small dilated blood vessels), and papules or pustules (Figure 1) (1). With its long-lasting and relapsing course, rosacea not only causes visible facial disfigurement but is also frequently accompanied by psychological comorbidities such as anxiety, depression, and social withdrawal, which together markedly impair patients’ quality of life (2, 3).

**Figure 1:**
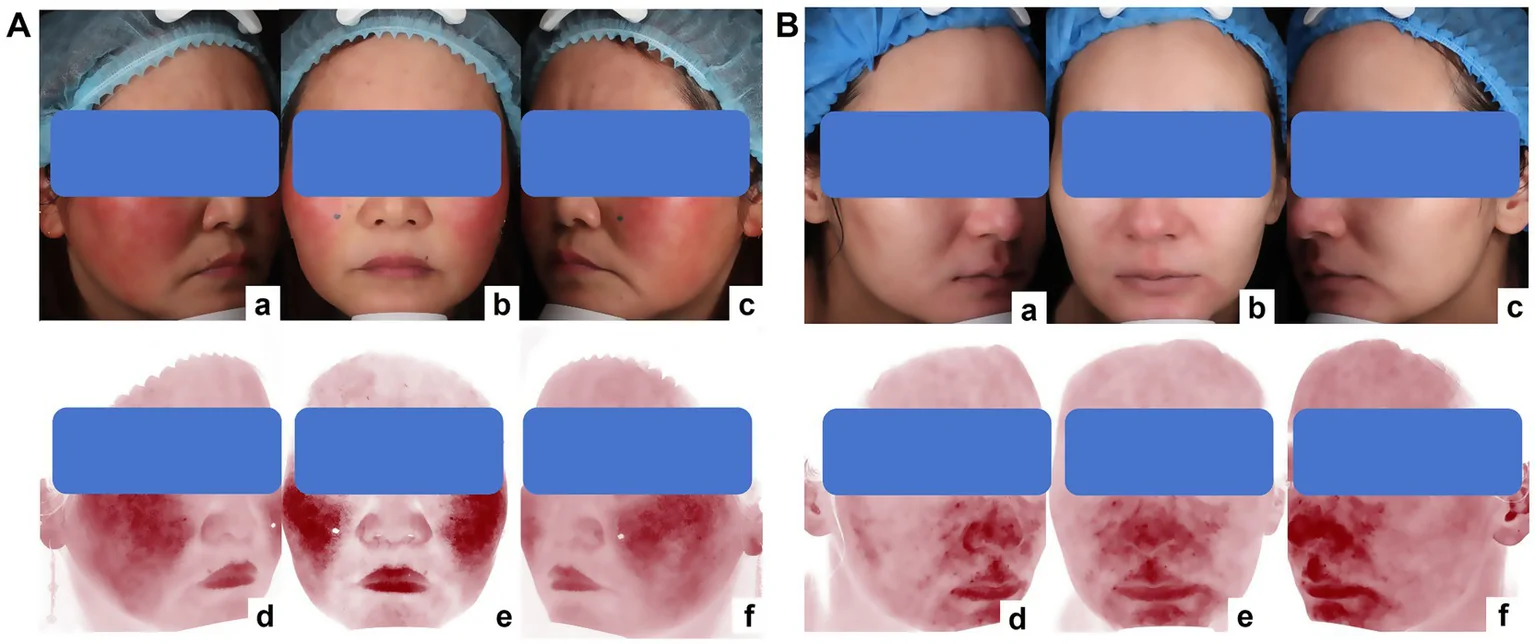
Clinical manifestations of rosacea **(A, B)**.

Effective management generally requires long-term, often multimodal approaches. Current options range from conventional topical or systemic medications to physical therapies, such as intense pulsed light (IPL), and more recently, emerging interventions like microdroplet botulinum toxin injections (1, 4, 5). Although these therapies can be effective, their high cumulative costs and the need for repeated sessions substantially increase the economic burden for patients. These financial challenges, in turn, represent major barriers to treatment choice, long-term adherence, and sustained improvements in daily functioning.

The emerging biopsychosocial model of medicine highlights the need for a comprehensive assessment of both the clinical manifestations and the broader burden of disease. Within this framework, health-related quality of life (HRQoL) has become a vital outcome metric for evaluating disease impact (6). Willingness to pay (WTP) is a widely used health economics measure that determines the maximum amount an individual is prepared to spend for specific health improvements, such as symptom relief or cure (7). In dermatology, WTP serves not only as an indicator of financial commitment but also as a proxy for the perceived value of treatment, encapsulating patients’ subjective appraisal of their health state and expectations for therapeutic benefit. This metric has been applied extensively for cost–benefit analyses and HRQoL assessments across a variety of health conditions (6–8). Although WTP has been investigated in skin disorders such as psoriasis in both domestic and international contexts (8), there is a marked lack of systematic research in rosacea populations, particularly within defined geographical and cultural settings.

Southwest China offers a unique case study, as local environmental and lifestyle factors—including a hot, humid climate, high ultraviolet exposure, and frequent consumption of spicy, oily foods—are recognized triggers or aggravators of rosacea (9, 10). These factors may influence disease expression, treatment preferences, and the perceived value of therapy compared to other regions. Therefore, exploring rosacea patients’ WTP in this setting is critical to informing region-specific clinical strategies and public health policies.

This study aimed to quantify WTP for rosacea treatment in Southwest China and to examine the socioeconomic and clinical factors associated with it. By adopting a patient-centered approach, we seek to generate evidence to inform equitable pricing, optimize service delivery, and strengthen health education—thus improving treatment adherence and facilitating tailored, resource-efficient management for this population.

## 2 Study participants and methods

### 2.1 Study design and participants

This cross-sectional survey was conducted from January 2024 to April 2025 in the outpatient dermatology clinic of a large tertiary hospital in Chengdu, Southwest China. Patients with a confirmed diagnosis of rosacea were consecutively enrolled using a convenience sampling approach.

Inclusion criteria were: (1) diagnosis of rosacea according to the 2017 International Consensus Criteria (11) and the Chinese Guidelines for the Diagnosis and Treatment of rosacea (2021 edition) (12); (2) age ≥18 years; (3) provision of written informed consent after receiving detailed oral and written explanations of the study aims; and (4) sufficient literacy to independently complete the questionnaire or to do so with assistance from a trained investigator.

Exclusion criteria were: (1) concurrent facial dermatoses (e.g., acne vulgaris, eczema) or systemic diseases (e.g., lupus erythematosus, dermatomyositis) that could affect facial appearance or influence treatment choices; and (2) cognitive or communication impairments that would preclude understanding or completion of the questionnaire.

This study involved human participants and questionnaire-based data collection. The protocol was approved by the Ethics Committee of Chengdu Second People’s Hospital (Approval No. 2023402) and was conducted in accordance with the Declaration of Helsinki. All participants provided written informed consent after being fully briefed on the purpose and content of the study.

### 2.2 Study instruments and data collection

Data were collected using a structured questionnaire comprising four sections, completed by participants on site with investigator support when needed.

#### 2.2.1 Demographic and socioeconomic characteristics

Information was obtained on participants’ age, sex, marital status, educational attainment, occupation, and individual average monthly income.

#### 2.2.2 Willingness to pay (WTP)

The WTP was assessed using the contingent valuation method, which presents respondents with a hypothetical treatment scenario and asks for the maximum amount they would pay for a specified health improvement (6). To reduce variation in how the scenario was interpreted, trained investigators read every participant the same standardized description before the WTP question. The hypothetical treatment was described as producing marked and sustained improvement in rosacea—clear reduction in facial redness and inflammatory lesions, reasonably stable symptoms, and minimal adverse effects—while explicitly noting that this did not imply a permanent cure. Participants were then asked, in an open-ended format, for the maximum amount in Chinese yuan (CNY) they would be willing to pay each month for such a treatment, together with their preferred treatment modality. No preset prices or fixed percentage options were offered. Monthly income was recorded separately, and WTP was derived as a continuous percentage of monthly income. Because monthly income serves as the denominator of this ratio, any association between income categories and the WTP outcome must be interpreted in light of this arithmetic dependency (see Sections 4.3 and 4.5). Separately from the open-ended monthly WTP question, participants also reported their anticipated acceptable cost for a single treatment session, categorized as <500 CNY, 500–2,000 CNY, or ≥2,000 CNY; this variable was used only as a candidate covariate and was not used to derive the WTP percentage.

At our center, rosacea treatment is relatively costly and paid almost entirely out of pocket; only a small proportion of oral medication costs—typically under 5% of the total—is reimbursed through basic medical insurance outpatient coverage. This payment context makes the income-share metric a meaningful reflection of the relative financial burden patients are prepared to accept.

For analysis, WTP was dichotomized into lower (≤25% of monthly income) and higher (>25%). In the absence of an established cut-off for high versus low WTP in rosacea, we adopted 25% as an affordability-informed threshold inspired by the catastrophic health expenditure literature, in which income-share thresholds of 10 and 25% are used under Sustainable Development Goal indicator 3.8.2 (13). This value also lay very close to the observed sample mean WTP (25.33%), so within our data it separates patients with relatively higher and lower stated WTP. The 10-percentage-point bands shown in Figure 2 were applied afterwards for display only and are distinct from this analytic cut-off.

**Figure 2:**
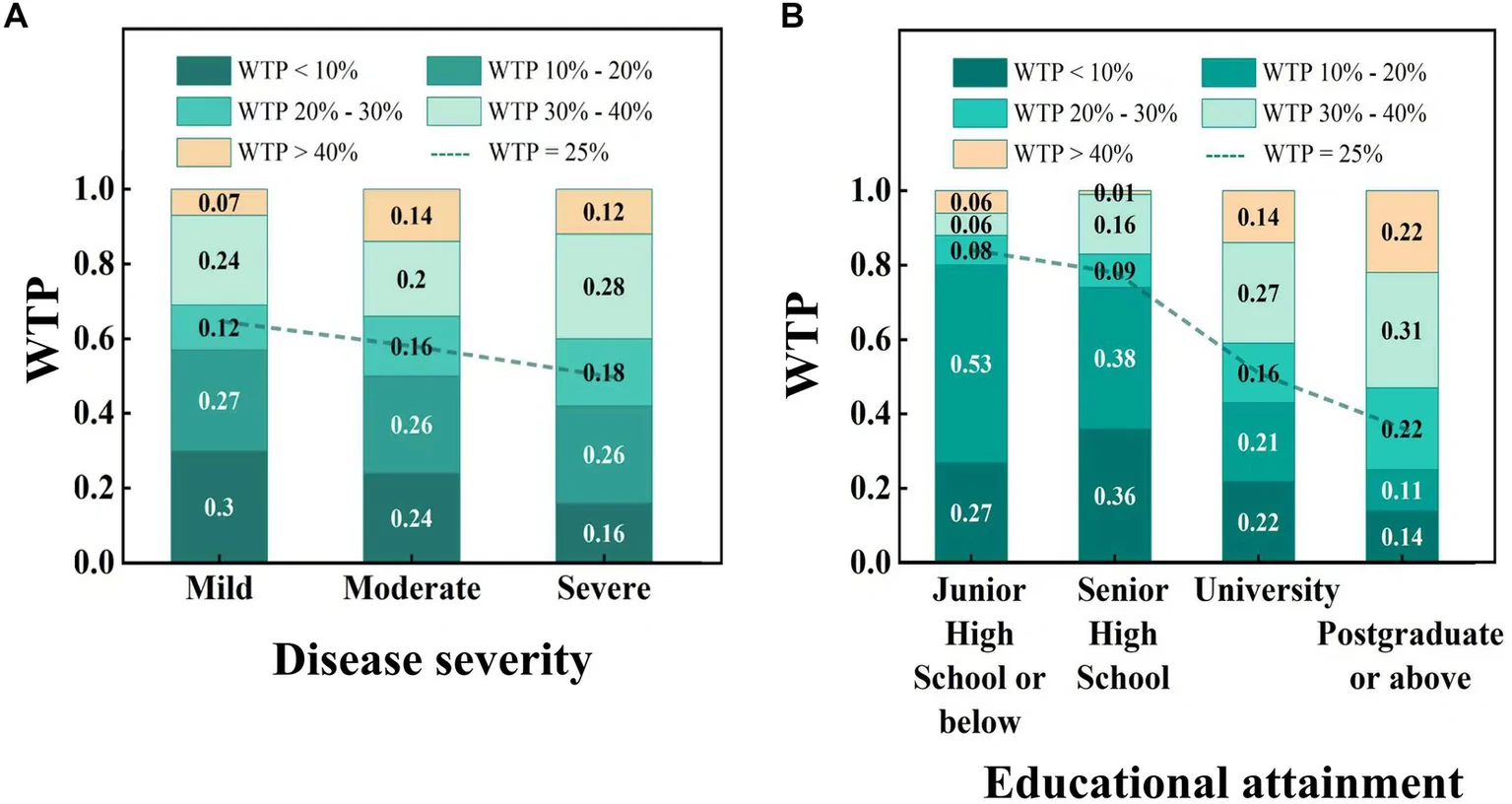
Distribution of willingness to pay (WTP), expressed as a percentage of monthly income and grouped into 10-percentage-point bands for display. **(A)** WTP by disease severity; **(B)** WTP by educational level. The vertical dashed line indicates the 25% analytic cut-off separating higher (>25%) from lower (≤25%) WTP; the display bands are descriptive only and distinct from this cut-off.

#### 2.2.3 Quality of life assessment

The validated Chinese version of the rosacea Quality of Life Questionnaire (RosaQoL) (14) was used to assess QoL impact during the past week. The instrument includes 19 items scored from 0 (never) to 4 (always), yielding a total score of 0–76; higher scores indicate greater impairment.

#### 2.2.4 Disease severity assessment

Disease severity was evaluated independently by experienced dermatologists using the clinical criteria in the Chinese Guidelines for the Diagnosis and Treatment of rosacea (2021 edition) (12), and categorized as mild, moderate, or severe.

### 2.3 Survey implementation

A dedicated survey team was established before the study began. All members completed standardized training covering the study protocol, questionnaire content, the fixed wording of the WTP scenario, and interviewing technique, to ensure consistency across investigators. Eligible patients were approached in the outpatient waiting area or a private consultation room, where investigators explained the study, obtained written informed consent, and read the standardized WTP scenario aloud before recording responses. Data were collected through an online questionnaire administered on site; each survey took about 10 min. Investigators remained available for clarification and checked each submission immediately after completion to minimize item-level missing data.

### 2.4 Statistical analysis

Statistical analyses were performed using SPSS Statistics, version 26.0 (IBM Corp., Armonk, NY, United States). Variable normality was assessed prior to analysis (Shapiro–Wilk test). Continuous variables with normal distribution were expressed as mean ± standard deviation (SD) and compared between two groups using independent samples t-tests. Categorical variables were described as frequencies and percentages and compared using chi-square tests or Fisher’s exact tests, as appropriate. The primary outcome for regression analysis was high WTP, defined as willingness to pay more than 25% of individual monthly income. Variables significant at *p* < 0.10 in univariate analyses were entered into a binary logistic regression model to identify factors associated with high WTP, with results reported as odds ratios (ORs) and 95% confidence intervals (CIs).

Because dichotomizing a continuous outcome may discard information and make results sensitive to the selected threshold, we performed a sensitivity analysis using ordinary least-squares (OLS) regression, with WTP as a continuous percentage of monthly income as the dependent variable and the full set of candidate demographic, socioeconomic, and clinical variables assessed in this study (age, sex, marital status, educational attainment, occupation, preferred treatment modality, anticipated single-treatment cost, monthly income, disease severity, comorbidity, and RosaQoL total score). This candidate set is slightly broader than that used in the primary logistic model, which excluded sex and comorbidity at the univariate screening stage (both *p* > 0.10); the OLS model therefore served as a fully adjusted robustness check under a continuous outcome specification.

Before model fitting, multicollinearity among candidate covariates was assessed using the variance inflation factor (VIF) and tolerance. Model calibration was evaluated with the Hosmer–Lemeshow test, explained variation with the Cox–Snell and Nagelkerke pseudo-*R*^2^, and discrimination with the area under the receiver operating characteristic curve (AUC). Because the analytic sample had no missing values for the main analytic variables, complete-case analysis was used and no imputation was performed. A two-tailed *p* < 0.05 was considered statistically significant.

## 3 Results

### 3.1 Demographic and clinical characteristics

A total of 545 questionnaires were distributed, of which 39 (7.2%) were excluded under predefined quality-control criteria—implausibly short completion time, straight-lining, or substantial missing values for key variables—leaving 506 valid questionnaires for analysis (effective response rate 92.8%). The final analytic sample of 506 participants had no missing values for the main analytic variables, so complete-case analysis was used without imputation.

The cohort comprised 461 females (91.1%) and 45 males (8.9%), with a mean age of 35.28 ± 9.56 years (range 22–58). Regarding disease severity, 32.0% had mild rosacea, 43.3% moderate, and 24.7% severe. The mean RosaQoL score was 54.53 ± 14.62, indicating a substantial quality-of-life impact. Detailed sociodemographic, clinical, and economic characteristics—including marital status, educational attainment, occupation, income, preferred treatment modality, and comorbidities—are presented in Table 1.

**Table 1:** General characteristics and univariate analysis of willingness to pay (WTP) among rosacea patients (*n* = 506).

Item	*N* (%)	Low WTP (%)	High WTP (%)	*χ* ^2^	*P*
Age group				13.338	0.004
20–30 years	198 (39.13)	94 (18.58)	104 (20.55)		
31–40 years	169 (33.40)	72 (14.23)	97 (19.17)		
41–50 years	92 (18.18)	56 (11.07)	36 (7.11)		
≥50 years	47 (9.29)	31 (6.13)	16 (3.16)		
Gender				–	0.378
Male	45 (8.89)	21 (4.15)	24 (4.74)		
Female	461 (91.11)	232 (45.85)	229 (45.26)		
Marital status				–	0.067
Unmarried	173 (34.19)	78 (15.42)	95 (18.77)		
Married	333 (65.81)	175 (34.58)	158 (31.23)		
Educational Level				53.42	<0.001
Junior High School or below	51 (10.08)	41 (8.10)	10 (1.98)		
Senior High School	77 (15.22)	57 (11.26)	20 (3.95)		
University/College	341 (67.39)	146 (28.85)	195 (38.54)		
Postgraduate or above	37 (7.31)	9 (1.78)	28 (5.53)		
Occupation				20.97	<0.001
Management Personnel	105 (20.75)	41 (8.10)	64 (12.65)		
Professional Technical Personnel	95 (18.77)	34 (6.72)	61 (12.06)		
Manual, Service, and Agricultural Workers	84 (16.60)	50 (9.88)	34 (6.72)		
Others: Retired or Unemployed	222 (43.87)	128 (25.30)	94 (18.58)		
Chronic Disease				–	0.104
No	448 (88.54)	219 (43.28)	229 (45.26)		
Yes	58 (11.46)	34 (6.72)	24 (4.74)		
Preferred Treatment Modality				7.024	0.030
Drug Treatment	207 (40.91)	118 (23.32)	89 (17.59)		
Drug Treatment + Phototherapy	116 (22.92)	54 (10.67)	62 (12.25)		
Comprehensive Treatment	183 (36.17)	81 (16.01)	102 (20.16)		
Expected Single Treatment Cost				5.286	0.071
<500 RMB	147 (29.05)	83 (16.40)	64 (12.65)		
500–2000 RMB	185 (36.56)	94 (18.58)	91 (17.98)		
≥2000 RMB	174 (34.39)	76 (15.02)	98 (19.37)		
Average Monthly Income (RMB)				18.47	<0.001
<5,000 RMB	232 (45.85)	140 (27.67)	92 (18.18)		
5,000–8,000 RMB	166 (32.81)	67 (13.24)	99 (19.57)		
≥8,000 RMB	108 (21.34)	46 (9.09)	62 (12.25)		
Disease Severity				6.52	0.038
Mild	162 (32.02)	92 (18.18)	70 (13.83)		
Moderate	219 (43.28)	109 (21.54)	110 (21.74)		
Severe	125 (24.70)	52 (10.28)	73 (14.43)		
RosaQoL Total Score	Mean ± SD	53.19 ± 14.21	55.87 ± 14.87	−2.06^	0.040

“–” indicates Fisher’s exact test was used, thus no Chi-square value. “^” indicates *t*-value from independent samples *t*-test.

### 3.2 Univariate analysis of factors associated with WTP

Significant statistical differences in the distribution of WTP were observed across age groups, educational level, occupation, preferred treatment modality, monthly income, and disease severity (all *p* < 0.05). In contrast, no significant statistical differences were found with respect to sex, marital status, anticipated single-session treatment cost, or presence of chronic disease (all *p* > 0.05), as shown in Table 1.

Further analysis indicated that WTP significantly varied by age group (*χ*^2^ = 13.338, *p* = 0.004): the proportion of participants with high WTP was greatest among the 20–40 year age group (20–30 years: 52.5%; 31–40 years: 57.4%), and lowest in those aged ≥50 years (34.0%). Educational attainment was strongly and positively associated with WTP (*χ*^2^ = 53.42, *p* < 0.001), with the proportion of high WTP rising from 19.6% among those with junior high school education or below to 75.7% among those with a postgraduate degree or above. Significant differences were also observed across occupational categories (*χ*^2^ = 20.97, *p* < 0.001): managers (61.0%) and professionals/technical staff (64.2%) exhibited higher rates of high WTP, whereas manual, service, and agricultural workers showed the lowest proportion (40.5%). Patients preferring combination or comprehensive treatment approaches had a significantly higher proportion of high WTP (53.4 and 55.7%, respectively) compared to those inclined toward medication therapy alone (43.0%) (*χ*^2^ = 7.024, *p* = 0.03). Monthly personal income was another significant factor (*χ*^2^ = 18.47, *p* < 0.001): the proportion of high WTP was markedly greater among those with monthly incomes ≥5,000 CNY (5,000–8,000 CNY: 59.6%; ≥8,000 CNY: 57.4%) compared to those earning <5,000 CNY (39.7%). Disease severity was also significantly correlated with WTP (*χ*^2^ = 6.52, *p* = 0.038), with the proportion of high WTP increasing stepwise from mild (43.2%) to moderate (50.2%) and severe cases (58.4%).

### 3.3 Multivariate analysis of factors associated with WTP

Variables with *p* < 0.1 in univariate analysis (age, marital status, educational attainment, occupation, preferred treatment modality, anticipated single-treatment cost, personal monthly income, and disease severity), together with the RosaQoL total score, were entered into a binary logistic regression model. Multicollinearity diagnostics did not indicate serious collinearity: VIFs ranged from 1.063 to 5.537 and tolerances from 0.181 to 0.941. For the variables of particular concern, VIFs were low: educational attainment, 1.459; income, 1.477; and occupation, 1.259. The model showed adequate calibration (Hosmer–Lemeshow *χ*^2^ = 4.466, df = 8, *p* = 0.813) and acceptable discrimination (AUC = 0.712, 95% CI: 0.667–0.756, *p* < 0.001; Nagelkerke pseudo-*R*^2^ = 0.180, Cox–Snell *R*^2^ = 0.135).

Educational attainment was independently associated with WTP, and pairwise contrasts suggested a severity-related gradient in WTP when mild and moderate disease were compared with severe disease (Table 2). Other factors—age, marital status, occupation, preferred treatment modality, anticipated single-treatment cost, personal monthly income, and RosaQoL score—showed no significant independent association after adjustment (*p* > 0.05).

**Table 2:** Binary logistic regression analysis of factors associated with high willingness to pay among patients with rosacea (*n* = 506).

Item	Variable	*B*	Std. Error	Wald	df	Exp(B)	95% CI (Lower)	95% CI (Upper)	*P*
Age group				1.921	3				0.589
	20–30 years	−0.071	0.448	0.025	1	0.931	0.387	2.242	0.874
	31–40 years	0.219	0.412	0.282	1	1.244	0.555	2.778	0.595
	41–50 years	−0.114	0.430	0.071	1	0.892	0.384	2.071	0.790
	≥50 years (Reference)					1.00			
Marital Status				0.090	1				0.764
	Unmarried	0.079	0.262	0.090	1	1.082	0.647	1.809	0.764
	Married (Reference)					1.00			
Educational Level				23.663	3				<0.001
	Junior High School or below	−2.083	0.588	12.558	1	0.124	0.039	0.394	<0.001
	Senior High School	−2.023	0.519	15.210	1	0.132	0.048	0.366	<0.001
	University/College	−0.817	0.418	3.811	1	0.442	0.195	1.003	0.051
	Postgraduate or above (Reference)					1.00			
Occupation				6.218	4				0.183
	Management Personnel	0.490	0.277	3.129	1	1.632	0.948	2.809	0.077
	Professional Technical Personnel	0.553	0.286	3.743	1	1.738	0.993	3.043	0.053
	Workers/Sales/Service Personnel	−0.191	0.518	0.136	1	0.826	0.299	2.279	0.712
	Agriculture/Animal Husbandry	0.451	0.330	1.861	1	1.569	0.821	2.999	0.173
	Others: Retired or Unemployed (Reference)					1.00			
Preferred Treatment Modality				0.337	2				0.845
	Drug Treatment	−0.012	0.292	0.002	1	0.988	0.557	1.753	0.968
	Drug Treatment + Phototherapy	0.135	0.264	0.263	1	1.145	0.682	1.922	0.608
	Comprehensive Treatment (Reference)					1.00			
				0.141	2				0.932
Expected Single Treatment Cost	<500 RMB	0.014	0.340	0.002	1	1.014	0.520	1.976	0.967
	500–2000 RMB	−0.070	0.260	0.072	1	0.932	0.560	1.553	0.788
	≥2000 RMB (Reference)					1.00			
Average Monthly Income (RMB)				2.823	2				0.244
	<5,000 RMB	−0.076	0.289	0.068	1	0.927	0.526	1.634	0.794
	5,000–8,000 RMB	0.314	0.272	1.331	1	1.369	0.803	2.334	0.249
	≥8,000 RMB (Reference)					1.00			
Disease Severity				5.318	2				0.070
	Mild	−1.276	0.609	4.389	1	0.279	0.085	0.921	0.036
	Moderate	−0.847	0.371	5.201	1	0.429	0.207	0.888	0.023
	Severe (Reference)					1.00			
Quality of Life	RosaQoL Total Score	−0.014	0.016	0.817	1	0.986	0.957	1.017	0.366

Model fit, Hosmer–Lemeshow *χ*^2^ = 4.466, df = 8, *p* = 0.813; Cox–Snell *R*^2^ = 0.135; Nagelkerke *R*^2^ = 0.180; AUC = 0.712 (95% CI, 0.667–0.756). Multicollinearity was acceptable (all VIF ≤ 5.54).

For educational attainment, compared with postgraduate or higher education (reference), patients with junior high school education or below (OR = 0.124, 95% CI: 0.039–0.394, *p* < 0.001), senior high school (OR = 0.132, 95% CI: 0.048–0.366, *p* < 0.001), and university/college education (OR = 0.442, 95% CI: 0.195–1.003, *p* = 0.051) had lower odds of high WTP.

For disease severity, with severe disease as the reference, both mild disease (OR = 0.279, 95% CI: 0.085–0.921, *p* = 0.036) and moderate disease (OR = 0.429, 95% CI: 0.207–0.888, *p* = 0.023) were associated with significantly lower odds of high WTP, indicating a severity-related gradient consistent with the univariate analysis. Clinically, severe rosacea typically involves more extensive erythema, a heavier inflammatory-lesion burden, and a greater likelihood of phymatous or disfiguring change—features that are more conspicuous, harder to treat, and require sustained long-term management, which may underlie the higher WTP. We note that the overall Wald test for severity was borderline (*p* = 0.070); therefore, we interpret this finding as an adjusted severity-related pattern supported by significant pairwise contrasts, while refraining from stronger claims about an overall independent effect or causality.

### 3.4 Distribution of WTP

Among the 506 patients, the mean maximum amount participants were willing to pay each month for marked and sustained improvement in rosacea was 1,664.72 ± 1,703.60 CNY, and the mean personal monthly income was 6,452.57 ± 1,719.30 CNY. The mean WTP—defined as the maximum proportion of monthly income participants would allocate to treatment—was 25.33% ± 22.27% (mean ± SD); the wide standard deviation reflects the broad spread of responses shown in Figure 2. These figures represent stated WTP rather than realized out-of-pocket expenditure.

Disease severity (*r* = 0.124, *p* < 0.01) and educational attainment (*r* = 0.28, *p* < 0.01) were both positively correlated with WTP. The distribution of WTP by disease severity and educational level is shown in Figure 2, where a vertical dashed line marks the 25% cut-off used to separate higher (>25%) from lower (≤25%) WTP.

### 3.5 Sensitivity analysis: continuous WTP outcome

In the OLS model with continuous WTP percentage as the dependent variable, the covariates jointly explained a small proportion of the variance (*R*^2^ = 0.082; adjusted *R*^2^ = 0.040). Although the overall model was statistically significant (F-test *p* = 0.006), no individual covariate reached statistical significance at the 0.05 level. Only two covariates showed borderline associations (*p* < 0.10): senior high school education (*B* = −9.46, *p* = 0.057) and management occupation (*p* = 0.086), both directionally consistent with the primary logistic model. All remaining covariates, including clinician-rated disease severity, absolute monthly income, and RosaQoL total score, had *p* > 0.10. Overall, these findings indicate attenuation of the associations observed in the primary dichotomous-outcome analysis when WTP was modeled as a continuous percentage.

## 4 Discussion

### 4.1 Rationale for measuring WTP and regional context

Rosacea is a chronic, disfiguring dermatosis that imposes substantial physical and psychological burdens, yet WTP—a useful proxy for the perceived value of treatment—has rarely been studied in this population. To capture the relative economic burden patients are prepared to accept, we expressed WTP as a proportion of monthly income.

This proportional metric was informed by, but should not be equated with, the concept of catastrophic health expenditure (CHE). CHE is defined by actual out-of-pocket payments, usually at the household level, with Sustainable Development Goal indicator 3.8.2 using income-share thresholds of 10 and 25% (13). Our measure differs in two important respects: it reflects stated willingness to pay for a hypothetical treatment rather than realized spending, and it is expressed against individual rather than household resources. We therefore treat 25% as an affordability-informed cut-off inspired by the CHE literature—reinforced by its proximity to the observed sample mean WTP—rather than as a marker of catastrophic burden, and our WTP estimates should not be read as actual expenditure.

Our 506 patients showed demographics consistent with known rosacea epidemiology (1), being predominantly young to middle-aged women (72.5% aged 20–40 years). Nearly half (45.9%) reported a monthly income below 5,000 CNY, reflecting the comparatively lower economic development of this region relative to more affluent eastern provinces; a proportional metric is therefore better suited than absolute amounts to capturing the relative priority assigned to rosacea treatment. The regional setting is also distinctive on biological grounds. Compared with national cohorts such as that of Huang et al. (15), our patients live in Southwest China, including the Sichuan Basin, where high humidity, enclosed basin topography with poor air circulation, and regionally relevant ultraviolet (UV) exposure may interact with lifestyle triggers. Chronic UV exposure is widely regarded as a key aggravator of rosacea, promoting dermal matrix degradation, vascular hyper-reactivity, and oxidative stress that reinforce the persistent erythema and telangiectasia central to the disease. A warm, humid microclimate may further favor proliferation of Demodex mites and associated innate-immune activation, while the regional preference for spicy, hot, and oily foods provides recurrent thermal and capsaicin-mediated vasodilatory stimuli that repeatedly trigger flushing (9, 10). Consistent with our cross-sectional design, we present these as background mechanisms supported by prior research rather than as causal inferences from our own data; nonetheless, the convergence of these triggers plausibly contributes to a higher and more recurrent disease burden in this region, which in turn makes patients’ willingness to invest in sustained, long-term treatment particularly relevant. Region-specific data therefore complement national surveys and support locally grounded counseling and affordability discussions.

### 4.2 Factors independently associated with WTP

After adjustment for multiple socioeconomic and clinical variables, educational attainment remained independently associated with higher WTP. Clinician-rated disease severity showed an adjusted severity-related pattern: patients with mild or moderate disease had significantly lower odds of high WTP than those with severe disease, although the overall Wald test for severity was borderline. Therefore, the severity finding is interpreted cautiously as being supported by pairwise contrasts rather than by a clearly significant overall effect.

#### 4.2.1 Educational attainment

Compared with patients holding a postgraduate or higher qualification, those with lower educational attainment were significantly less likely to report high WTP (*p* < 0.05), consistent with research linking education to health behaviors and WTP in other chronic conditions (16, 17). Educational attainment is best understood here as a broad sociodemographic indicator. It may partly reflect health literacy and the ability to access, interpret, and appraise health information (18), but it can equally capture socioeconomic position, employment security, aesthetic preferences, and prior healthcare experience, which our data cannot separate. Importantly, the association persisted after adjustment for monthly income, indicating that it is not simply a matter of ability to pay; however, because we did not administer a validated health literacy instrument, we cannot conclude that health literacy is the operative mechanism.

This pattern nonetheless has practical value. Tailoring health education to patients’ educational background—using accessible, visually rich materials, personalized face-to-face explanation, and new-media platforms—may help patients with lower educational attainment understand the chronic, relapsing nature of rosacea and the rationale for sustained, standardized treatment, supporting more informed appraisal of its value.

#### 4.2.2 Disease severity

Clinician-rated disease severity showed a severity-related association with WTP, with patients with severe rosacea having higher odds of high WTP than those with mild or moderate disease. Although the Health Belief Model emphasizes perceived rather than clinician-rated severity, more visible and extensive clinical manifestations may contribute to patients’ perception of disease seriousness and thereby influence treatment-seeking and payment decisions (19, 20). Severe rosacea presents with more pronounced erythema and inflammatory lesions and is frequently accompanied by burning or stinging discomfort, social dysfunction, and psychological distress related to facial disfigurement (2, 3), features that plausibly heighten the perceived value of effective treatment.

By contrast, the RosaQoL total score, although documenting the considerable quality-of-life impairment in our cohort (mean 54.53), was not independently associated with high WTP after multivariable adjustment. We therefore refrain from presenting subjective disease burden as a driver of WTP. Taken together, these findings suggest that, in this population, stated WTP was more closely related to clinician-rated clinical severity than to the RosaQoL total score after adjustment. Clinically, this supports assessing both the visible disease burden and its subjective impact when discussing treatment options, while recognizing that the two may relate differently to patients’ willingness to pay.

### 4.3 Interpretation of multivariate findings and WTP metric validity

In univariate analyses, age, occupation, preferred treatment modality, and personal monthly income were associated with WTP; however, these associations were attenuated and no longer statistically significant after multivariable adjustment for educational attainment, clinician-rated disease severity, and RosaQoL score. Importantly, our findings suggest a divergence between objective and subjective disease measures. In pairwise contrasts, patients with mild or moderate disease had lower odds of high WTP than those with severe disease (OR = 0.28–0.43, reference category: severe; *p* < 0.05), whereas the overall Wald test for severity was borderline and the RosaQoL total score was not significant after adjustment (*p* = 0.366).

This pattern indicates that, in our outpatient cohort from Southwest China, WTP was more strongly associated with clinician-rated severity than with the patient-reported RosaQoL total score. Compared with prior work such as Huang et al. (15), which used the generic DLQI, our use of the rosacea-specific RosaQoL provides a more targeted assessment of disease-related quality of life and helps clarify that the statistical association with WTP in our model appears to be driven primarily by clinician-rated severity rather than overall HRQoL score.

Notably, personal monthly income was not independently associated with high WTP in the primary logistic model. However, because monthly income is embedded in the denominator of the WTP percentage, this null finding is partly a function of the metric’s construction and should not be interpreted as evidence that income is irrelevant to treatment affordability.

### 4.4 Conclusion and implications for practice

To our knowledge, this is the first systematic investigation of WTP for rosacea treatment in Southwest China incorporating RosaQoL into multivariate modeling. Educational attainment was the only factor to reach overall significance in the primary logistic model. In the OLS sensitivity analysis using continuous WTP percentage, this association was attenuated to non-significance, with only the senior high school contrast remaining borderline (*p* = 0.057) and directionally consistent. Clinician-rated disease severity likewise showed attenuation in the continuous-outcome model. Taken together, these findings suggest that the observed associations were sensitive to how WTP was specified and therefore should be interpreted cautiously. This difference is not unexpected because the logistic model estimates the probability of exceeding the 25% threshold, whereas the OLS model examines mean variation across the full continuous WTP distribution.

These findings have direct relevance for shared decision-making, because in our setting proactive maintenance therapy is largely paid out of pocket and some internationally used agents are not routinely available in mainland China; for example, the 2021 Chinese guideline explicitly notes that topical ivermectin cream is not marketed in China (12). Patients therefore often bear sustained personal expenditure to maintain disease control. In this context, the aim of incorporating WTP into care is not to increase spending but to support informed, affordability-sensitive counseling and adherence to clinically appropriate, guideline-based treatment. Because educational attainment was associated with WTP independently of income, we suggest integrating education-sensitive communication strategies—such as visualized disease-course charts and clear comparisons of maintenance versus acute treatment—into routine counseling, helping patients, particularly those with lower educational attainment, understand the value of sustained therapy and make informed choices within their means.

### 4.5 Strengths and limitations

This study has several limitations. First, the sample was drawn from a single hospital-based outpatient population in a city with high uptake of aesthetic services, and women were overrepresented (91.1%); this may introduce selection bias and limit generalizability, particularly to men, who may underuse dermatology services. Multi-center, community-based studies are needed to confirm whether these patterns hold more broadly. Second, the WTP scenario described marked and sustained improvement but was not tied to a quantitative, time-bound endpoint (for example, ≥75% reduction in erythema and inflammatory lesions sustained for 6 months); a more precisely specified outcome would further reduce variation in interpretation. Third, our income-share WTP reflects stated preference against individual income and cannot be equated with household-level catastrophic health expenditure. Moreover, because income is the denominator of this metric, the null finding for absolute income is partly shaped by the metric’s construction rather than being a wholly independent result. Future work should incorporate actual out-of-pocket expenditure data. Fourth, the primary analysis dichotomized a continuous WTP percentage at 25%, a threshold close to the sample mean. Although this cut-off was selected as an affordability-informed benchmark, dichotomization may discard information, reduce precision, and make findings sensitive to the chosen threshold. Consistent with this concern, in the sensitivity analysis using continuous WTP percentage as the outcome, no individual covariate remained statistically significant at *p* < 0.05. Therefore, the associations observed in the primary analysis should be interpreted cautiously and regarded as potentially sensitive to outcome specification. Fifth, we did not administer a validated health literacy instrument, so we cannot determine whether health literacy mediates the association between education and WTP; future studies should include such measures alongside indicators of socioeconomic position. Sixth, the postgraduate group used as the reference category for educational attainment was small (*n* = 37), and some occupational categories were sparsely populated (e.g., workers/sales/service personnel, *n* = 26). These small cells likely contributed to the wide confidence intervals for several estimates, particularly the education-related odds ratios, so the magnitude of the education–WTP association should be interpreted with caution. Reassuringly, the overall model still included 253 high-WTP events for approximately 20 estimated parameters (about 12.6 events per parameter), exceeding the commonly cited minimum of 10. Finally, individual health-insurance status was not recorded as a separate variable; although rosacea care at our center is almost entirely out of pocket, future studies should systematically collect insurance and reimbursement information.

## Data Availability

The datasets presented in this article are not readily available because the raw dataset generated and analyzed during this study is not publicly available due to privacy and ethical restrictions. No additional data is shared for this research. Requests to access the datasets should be directed to Hongye Wu, wuwuwumail@163.com.

## References

[1] Shi C Ding Y Tao C Peng Y Li W Yi Y . Interpretation of the 2021 British Association of Dermatologists guidelines for the management of rosacea. Chin J Evid Based Med. (2022) 22:134–9. doi: 10.7507/1672-2531.202110153

[2] Seetan K Gablan M Alnaimi M Alhazaimeh D Younes MB Alnaimi A . Assessment of depressive and anxiety symptoms and health-related quality of life in Rosacea patients: a case-control study. Dermatol Res Pract. (2024) 2024:5532532. doi: 10.1155/drp/5532532, 39720781 PMC11668539

[3] Dai R Lin B Zhang X Lou Y Xu S. Depression and anxiety in Rosacea patients: a systematic review and Meta-analysis. Dermatol Ther. (2021) 11:2089–105. doi: 10.1007/s13555-021-00613-w, 34657997 PMC8611151

[4] Tong Y Luo W Gao Y Liu L Tang Q Wa Q. A randomized, controlled, Split-face study of botulinum toxin and broadband light for the treatment of Erythematotelangiectatic Rosacea. Dermatol Ther. (2022) 35:e15395. doi: 10.1111/dth.15395, 35187781

[5] Yang X Ouyang Y Deng Y Xiao Y Tang Y Jian D . Willingness-to-pay and benefit-cost analysis of botulinum toxin for the treatment of Rosacea in China: findings from a web-based survey. Patient Prefer Adherence. (2021) 15:1197–205. doi: 10.2147/PPA.S311322, 34113082 PMC8185460

[6] Xiao Y Chen L Jing D Shen M. Application and significance of willingness-to-pay in measuring the burden of skin diseases. Chin J Dermatol. (2019) 52:286–9. doi: 10.3760/cma.j.issn.0412-4030.2019.04.015

[7] Deng Q Liu F Shi W Xie H Li J Huang Y . Willingness to pay for treatment of rosacea and its influencing factors: a survey of patients in Changsha region. Chin J Dermatol. (2021) 54:352–5. doi: 10.35541/cjd.20200794

[8] Shen M Xiao H Zhu W Lu Y Jing D Kuang Y . Comparing willingness-to-pay and time-trade-off as the measures of burden of psoriasis: a pooled analysis of community, hospital, and web-based samples in China. J Dermatol. (2022) 49:1131–8. doi: 10.1111/1346-8138.16522, 35906766

[9] Zang R Yang J. Research progress in systemic treatment of rosacea. Chin J Dermatol Venereol. (2025) 39:697–703.

[10] Tang R. M. Geographical Characteristics of Southwestern Chinese Food Culture and its Application in Local Geographical Teaching (Master’s thesis, Huazhong Normal University), (2021)

[11] Galadari AR Darwish BR Kalia CS Mannis M Steinhoff M Tan J . Standard classification and pathophysiology of rosacea: the 2017 update by the National Rosacea Society expert committee. J Am Acad Dermatol. (2018) 78:148–55. doi: 10.1016/j.jaad.2017.08.037 29089180

[12] Rosacea Research Center of Chinese Society of Dermatology, Rosacea Professional Committee of Chinese Dermatologist Association. Chinese rosacea diagnosis and treatment guidelines (2021 edition). Chin J Dermatol. (2021) 54:279–88. doi: 10.35541/cjd.20201078

[13] World Health Organization. Global Monitoring Report on Financial Protection in Health 2021. Geneva: World Health Organization (2021).

[14] Deng Y Peng Q Yang S Jian D Wang B Huang Y . The Rosacea-specific quality-of-life instrument (RosQol): revision and validation among Chinese patients. PLoS One. (2018) 13:e0192487. doi: 10.1371/journal.pone.0192487, 29489857 PMC5831031

[15] Huang Y Ding Y Li S Wang B Zhao Z Huang Y . Health related quality of life of rosacea patients in China assessed by dermatology life quality index and willingness to pay. Patient Prefer Adherence. (2022) 16:659–70. doi: 10.2147/PPA.S345258 PMC8910444 35283624

[16] Cutler DM Lleras-Muney A. Understanding differences in health Behaviors by education. J Health Econ. (2010) 29:1–28. doi: 10.1016/j.jhealeco.2009.10.003, 19963292 PMC2824018

[17] Gu WY Jiang MZ Shen X Xiong JY. Study on preferences and willingness to pay for chronic disease medical-prevention integrated services among chronic disease patients based on the best-worst scaling method. Chin J Soc Med. (2022) 39:720–4.

[18] Khor CY Hong M. Health literacy in mobile health applications: a choice modeling study. J Consum Health Internet. (2025) 29:208–49. doi: 10.1080/15398285.2025.2497743

[19] Rosenstock IM. Historical origins of the health belief model. Health Educ Monogr. (1974) 2:328–35. doi: 10.1177/109019817400200403

[20] Janz NK Becker MH. The health belief model: a decade later. Health Educ Q. (1984) 11:1–47. doi: 10.1177/109019818401100101, 6392204

